# Electrochemical DNA-nano biosensor for the detection of Goserelin as anticancer drug using modified pencil graphite electrode

**DOI:** 10.3389/fonc.2024.1321557

**Published:** 2024-05-01

**Authors:** Layla Abd-Al-Sattar Sadiq Laylani, F. Al-dolaimy, Ali Altharawi, Ghasen M. Sulaman, Mohammed Ahmed Mustafa, Adnan Taan Alkhafaji, Ali G. Alkhatami

**Affiliations:** ^1^ Community Health Department, Kirkuk Technical Institute, Northern Technical University, Mosul, Iraq; ^2^ Community Health Department, Al-Zahraa University for Women, Karbala, Iraq; ^3^ Department of Pharmaceutical Chemistry, College of Pharmacy, Prince Sattam Bin Abdulaziz University, Al-Kharj, Saudi Arabia; ^4^ Department of Medical Laboratories, Sawa University, Almuthana, Iraq; ^5^ Department of Medical Laboratory Technology, University of Imam Jaafar AL-Sadiq, Baghdad, Iraq; ^6^ Cardiology Department, College of Medicine, Al-Ayen University, Thi-Qar, Iraq; ^7^ Department of Clinical Laboratory Sciences, College of Applied Medical Sciences, King Khalid University, Abha, Saudi Arabia

**Keywords:** Goserelin, carbon nanotubes, copper oxide, DNA biosensor, Voltammetry

## Abstract

Goserelin is an effective anticancer drug, but naturally causes several side effects. Hence the determination of this drug in biological samples, plays a key role in evaluating its effects and side effects. The current studies have concentrated on monitoring Goserelin using an easy and quick DNA biosensor for the first time. In this study, copper(II) oxide nanoparticles were created upon the surface of multiwalled carbon nanotubes (CuO/MWCNTs) as a conducting mediator. The modified pencil graphite electrode (ds-DNA/PA/CuO/MWCNTs/PGE) has been modified with the help of polyaniline (PA), ds-DNA, and CuO/MWCNTs nanocomposite. Additionally, the issue with the bio-electroanalytical guanine oxidation signal in relation to ds-DNA at the surface of PA/CuO/MWCNTs/PGE has been examined to determination Goserelin for the first time. It also, established a strong conductive condition to determination Goserelin in nanomolar concentration. Thus, Goserelin’s determining, however, has a 0.21 nM detection limit and a 1.0 nM-110.0 µM linear dynamic range according to differential pulse voltammograms (DPV) of ds-DNA/PA/CuO/MWCNTs/PGE. Furthermore, the molecular docking investigation highlighted that Goserelin is able to bind ds-DNA preferentially and supported the findings of the experiments. The determining of Goserelin in real samples has been effectively accomplished in the last phase using ds-DNA/PA/CuO/MWCNTs/PGE.

## Introduction

LHRH (luteinizing hormone-releasing hormone) analogue Goserelin is a parenterally administered synthetic decapeptide (1). Goserelin, buserelin, leuprolide, and triptorelin are examples of synthetic LHRH analogues that operate as agonists to activate the pituitary gland. LHRH analogues initially stimulate the pituitary gland’s production of luteinizing hormone (LH). But with continued dosing, the pituitary gland becomes desensitized, the amount of vacant LHRH receptors diminishes, and ultimately, the amount of LH secreted is decreased. The reduction in LH causes the blood levels of testosterone and estradiol in males and females, respectively, to drop to post-menopausal or castration levels (2).

Individuals with hormone-sensitive breast and prostate malignancies, along with those with a number of benign gynecological illnesses, such as endometriosis, endometrial thinning and uterine fibroids are treated with Goserelin (3). Continued administration of Goserelin induces a decrease in testosterone serum level below the castration threshold secondary to pituitary desensitization (4, 5). With this characteristic of Goserelin, the assessment of pharmacokinetics and pharmacodynamics is particular importance to assure the desired biological effects.

Radioimmunoassays (RIAs) have been used in Goserelin pharmacokinetic investigations and have been proven to be sensitive and selective (6). This method’s limit of detection (LOD) has been about 0.15 ng/mL. However, this is constrained by the cross-reactivity with peptides that share structural similarities, which obstructs the accurate quantification of Goserelin in plasma. Despite the fact that there are several analytical techniques for the quantitative or qualitative Goserelin determination, such as liquid chromatography–electrospray ionization mass spectrometry (LC–ESI-MS), radioimmunoassay, capillary zone electrophoresis (CZE)-UV/MS, CE hydrogen deuterium exchange-MS (CE-H/D-MS), multiple-injection CZE (MICZE) and quadrupole time-of-flight MS (Q-TOF MS), as well as fast atom bombardment-MS (FAB-MS) (7–13), these studies have often focused on medicinal formulations, degradation products, and crude synthesized peptide combinations. All of these present methods are not employed for the determination of Goserelin in a possible pharmaceutical dosage form and usually employed to quantify illegal substances in biological fluids. Therefore, a more precise, quick, and sensitive analytical methodology in measuring Goserelin of plasma still has to be created and validated.

Electrochemical methods have attracted a lot of interest in this arena because of their excellent sensitivity, selectivity, and capacity to reduce interferences (14–17). Because the electrochemical oxidation of the analyte often entails a significant overpotential at the surface of the bare electrodes, additional oxidizable substances frequently cause interference with the analyte’s identification (18–23). Chemically modified electrodes (CMEs) have gained significant attention because of their ability to transport electrons quickly, reduce overpotential with little surface fouling, and lower the overpotential of oxidizing species in order to avoid these interferences (24–28).

DNA biosensors are a type of biosensor that use DNA as the sensing element (29). DNA is subjected to chemical modifications through interactions with small molecules or reduction/oxidation pathways (30). In general, one can classify the drugs acting on DNA as follows: intercalative, groove binding and electrostatic mode (31). To design new drugs, many researchers have focused on DNA as the main cellular target. One of the key advantages of DNA biosensors for drug detection is their high sensitivity and selectivity (32). DNA biosensors can detect very low concentrations of drug with a high degree of accuracy, making them an ideal tool for monitoring drug levels in human serum samples. Another advantage of DNA biosensors is their ease of use and low cost (33).

Copper oxide (CuO) as a p-type semiconductor with a 1.2 eV band gap benefits in CMEs, including its low cost, non-toxicity, simple manufacturing process, ease of storage, and highly precise capacitances, have been well documented by researchers (34, 35). CuO may be created in a variety of nanostructures, including nanoflowers, nanorods, nanosheets, nanoneedles, nanospheres, nanourchins, and nanocubes, to improve its electrocatalytic characteristics (36). Another method involves mixing CuO with a few strong conductive substances like gold (Au), silver (Ag) and carbon nanotubes (CNTs), as well as graphene (37–41).

Carbon nanotubes (CNTs) have been extensively used as modifier in electroanalysis because of their strong electrical conductivity, wide surface area, chemical stability, high mechanical strength, easily chemically changeable surface and high surface/volume ratio (42). CNTs also make analyte oxidation easier. CuO will likely be integrated into CNTs to create a hybrid nanostructure for bio-electrochemical sensors. Recent research has concentrated on using CNTs with varied CuO morphologies to improve the characteristics of sensors (43). However, the limitations of its restricted liner range, poor sensitivity, low upper detection limit, and prolonged reaction time, as well as complex preparation methodologies have hampered its development and large-scale applications (44).

In this work, a pencil graphite electrode (PGE) has been modified with polyaniline (PA), CuO/MWCNTs nanocomposite, along with ds-DNA in order to develop a sensitive and focused biosensor for the measurement of Goserelin, given the significance in analyzing Goserelin and with the help of nanostructured DNA biosensors’ promise. The DNA biosensor’s sensitivity for measuring Goserelin was increased by modifying PGE with PA and CuO/MWCNTs nanocomposite. The ds-DNA/PA/CuO/MWCNTs/PGE assay, on the other hand, demonstrated to be a potent instrument for detecting Goserelin within injectable and urine samples. Additionally, docking simulations have been utilized to look at how DNA and Goserelin interact with one another.

## 2 Experimental

### 2.1 Chemicals and devices

Microwave oven (PANASONIC NN-GF352M, 1000 W, 2450 MHz) was used to synthesis of nanocomposite. Numerous methods were used to examine the microstructures and morphologies of the nanocomposite that this investigation manufactured. Scanning electron microscopy (SEM, Hitachi SU8010) working at 15 kV equipped energy dispersive X-ray spectroscopy (EDS) was used to observe the characteristics of the nanoparticles, and glancing angled (0.5°) X-ray diffraction (XRD, Philips X’pert MRD pro) with Cu K radiation (λ = 1.5418 Å) from 20 to 80° was used to analyze their crystal structures. The PH values were determined using a metrohm 710 pH meter. The UV-Vis absorption spectra were documented via an Analytik Jena SPECORD S100 UV-Vis spectrophotometer. Reversed-phase high-performance liquid chromatography experiments were performed on an Agilent 1200 series high performance liquid chromatograph (Agilent, USA) with a diode array detector.

Utilizing the CHI1030C Electrochemical Workstation, the electroanalytical experiments have been conducted utilizing electrochemical impedance spectroscopy (EIS), cyclic voltammetry (CV), and differential pulse voltammetry (DPV). Z-VIEW software used to calculate Rct value. At 25 ± 1°C, the application was made to a conventional cell with three electrodes. The modified and unmodified PEG, a platinum wire, as well as the Ag/AgCl electrode were regarded as the working, auxiliary, along with reference electrodes, respectively. A frequency ranges between 0.1 and 105 Hz and an amplitude of 5 mV were used for the EIS research. By using DPV at a voltage of 0–1.0 V, the quantitative analysis and electrochemical behavior of Goserelin have been assessed.

Goserelin (>99.0%), dsDNA (calf thymus) (>99.0%), copper(II) acetate monohydrate (Cu(CH_3_COO)_2_H_2_O, >99.0%) and sodium hydroxide (>99.0%) with very high purity belonged to Merck Company. We bought MWCNTs (>90%; d = 70-110 nm, l = 5-9 μm) from Sigma-Aldrich. The MWCNTs were heated for 22 hours at reflux in a solution of 3.0 mol/L HNO_3_ and 2.0 mol/L H_2_SO_4_ (3:1, V/V) to eliminate all of the metal oxide present. The MWCNTs have been neutralized, cooled, and also dried at ambient temperature after being rinsed many times with distilled water.

### 2.2 Synthesis of CuO/MWCNT nanocomposite

40 mg of MWCNT and 20 mg of copper(II) acetate have been blended in a conventional mixing procedure, and crushed in a mortar and pestle. The MWCNT has been employed as a microwave absorbing substance and heating layer for facilitating copper(II) acetate decomposition. Such a situation results in forming CuO nanoparticles during microwave irradiation because of the influential microwave absorption of MWCNT (45). Final powder mix has been transported to a glassy vial and immediately inserted in the microwave oven for 20 min. According to the previous studies copper(II) acetate monohydrate decomposes during heating in two stages: I) (25-225°C) copper(II) acetate monohydrate dehydrates giving rise to copper(II) acetate, and II) (225-525°C) copper(II) acetate decomposes to CuO through complex oxidation reactions of Cu and Cu_2_O, simultaneously (46).

### 2.3 Fabrication of the biosensor and determination of loading amount of ds-DNA

The bare graphite pencil electrode has been placed in the solution of 0.1 M acetate buffer solution, cleaned with a sonic cleaner for five minutes, then submerged in a 0.5 M HCl solution and rinsed using 35 potential cycles between 0.0 and 1.2 V. Then, the PGE has been modified via electropolymerization in 100 mL of a 0.2 M aniline solution in water that also included 5.0 mg of CuO/MWCNTs nanocomposite. In order to achieve this, potentials between -0.3 and 1.0 V (40 cycles; cyclic voltammetry) were applied at a speed of 50 mV s^−1^.

The sensor has been submerged in ds-DNA solution (100.0 mg L^−1^) in the acetate buffer solution (ABS, 0.5 M, pH = 4.8) for almost 10 minutes, with the last stage being the application of a +0.50 V potential to the sensor.

The amount of ds-DNA immobilized on the modified electrode (Q, nmol mg-1), Equation 1, is obtained using the following equation via UV-Vis spectroscopy (47).


(1) $$\text{Q}=(\text{C}-{\text{C}}_{e})\text{V}/\text{m}$$

Where C and Ce, present the primarily (0.75 μM) and final ds-DNA concentration (0.38 μM), respectively, V is volume of ds-DNA solution (25.0 mL), and m is the weight of PA/CuO/MWCNT nanocomposite (2.43 mg). It should be mentioned that the final ds-DNA concentration upon immobilization was determined using an appropriate calibration curve. Based on the experimental section and obtained optimum ratio, the conjugation amount of ds-DNA onto the PA/CuO/MWCNT nanocomposite is calculated to be 3.81 μmol/mg.

### 2.4 Preparation of real specimens

The injectable Goserelin drug solution (labeled 10.8 mg/1 mL) was used as real sample without any pretreatment.

Blood samples gathered from healthy individuals in heparinized test tubes underwent a centrifugation for 10 min at 3000 rpm to separate the plasma, which was then refrigerated for next testing. Acetonitrile was used to deproteinate the plasma samples, so that the acetonitrile (2 mL) was appended to plasma and the mixture underwent a centrifugation for 10 min at 2000 rpm. The supernatant was diluted 50 times with ABS and poured into the voltametric cell (20 mL) for analysis with no need for pretreatment. The Goserelin was quantified in the blood samples according to standard addition method.

Urine specimens sampled from healthy subjects were analyzed to determine possible traces of Goserelin. After that, the samples were diluted 50 times with ABS to avoid the matrix effect of valid samples. The Goserelin was quantified in the urine samples according to standard addition method.

The interaction time in this section is the same as discussed in Section 3.4 (7 min). Standard addition involves adding known amounts of analyte to an unknown sample, a process known as spiking. By increasing the number of spikes, the analyst can extrapolate for the analyte concentration in the unknown that has not been spiked.

### 2.5 Molecular docking

The crystal structures of DNA hexamer d(CGATCG)2 sequence featuring PDB ID of 1Z3F, were employed. The AutoDock 4.2 software has been utilized to perform flexible ligand docking calculations utilizing the Lamarckian genetic algorithm with local search and empirical free energy function (48). With a grid-point spacing of 0.375 Å, the grid map has been intended to be sufficiently large (80 Å ×80 Å ×80 Å) to allow the Goserelin to revolve with ease. After eliminating the water molecules off the crystal structure, Gasteiger charges and any remaining hydrogen atoms were then added in the AutoDock Tools to form the DNA. All docking settings were set to default levels, with the exception of the number of docking runs that has been set to 200 and included 25,000,000 energy assessments for every run. Also, the most likely binding mode was chosen to be the docking conformation with the lowest binding energy (49).

## 3 Results and discussion

### 3.1 Characterizing CuO/MWCNT nanocomposite

**Figure 1:**
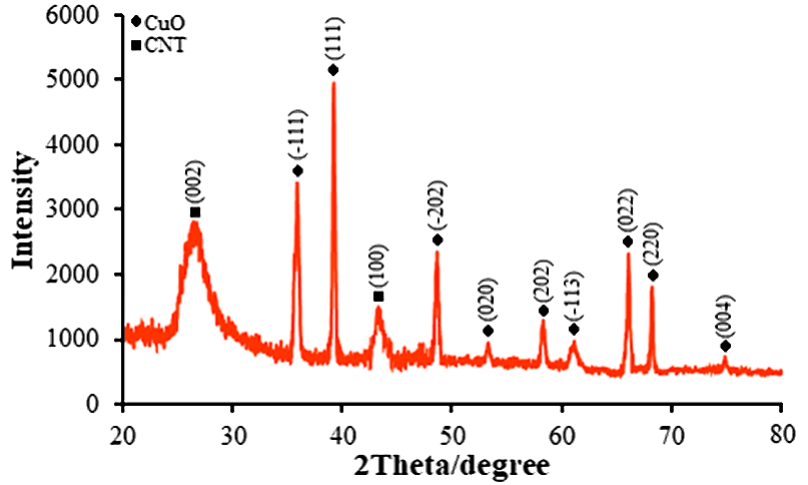
XRD pattern of CuO/MWCNTs nanocomposite.

CuO/MWCNT nanocomposite’s XRD patterns are depicted in **Figure 1**. The XRD outcomes are in line with typical CuO diffraction patterns (JSPDS NO. 80-1268) (50). The CuO/MWCNT nanocomposite that was created indicated crystallinity by sharp diffraction peaks. MWCNT’s XRD pattern showed two major peaks, located at 26.53° and 43.37°. CuO has been shown to exhibit obvious peaks at 2 positions of 35.93°, 39.20°, 48.67°, 53.25°, 58.34°, 61.10°, 66.21°, 68.35°, and 79.74°, respectively. These positions correspond to the lattice parameters of the pure phase of (-111), (111), (020), (202), (-113), (022), (220), and (004). Most of the CuO/MWCNT hybrids’ XRD peaks were nearly identical to those of the CuO sample, with a few small variations in peak attributes, such as peak width and peak intensity.

**Figure 2:**
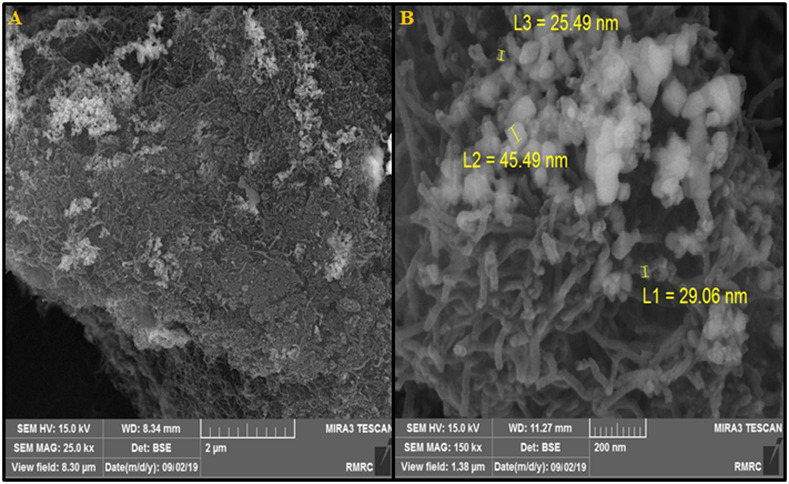
**(A)** FESEM image of CuO/MWCNTs nanocomposite **(B)** High resolution FESEM image of CuO/MWCNTs nanocomposite.

The influence of the microwave power on the product morphologies was analyzed by field emission scanning electron microscopy (FESEM). According to results, the MWCNT surface is fully and uniformly covered with CuO nanoparticles (**Figure 2**).

**Figure 3:**
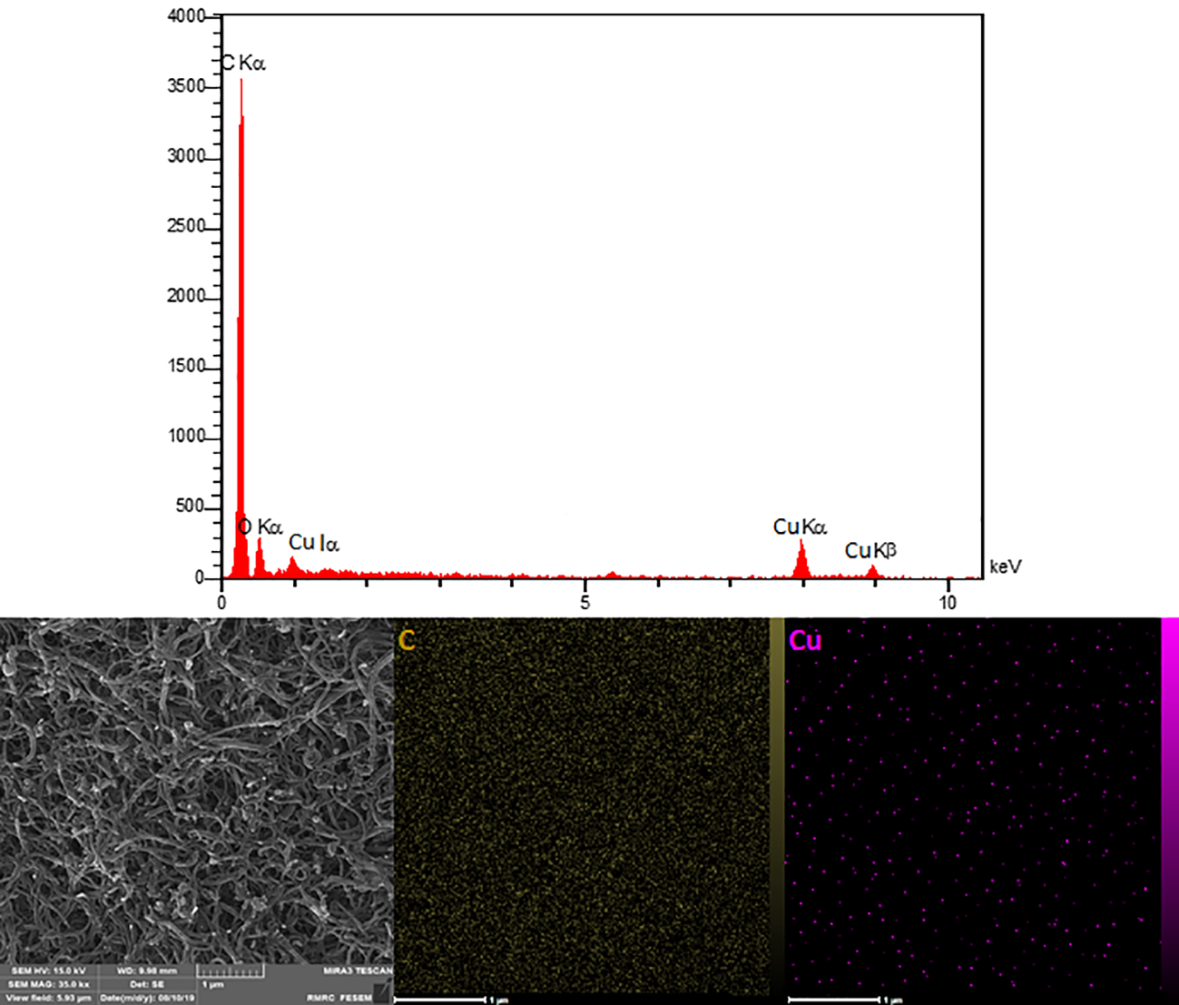
EDX spectra and elemental mapping of CuO/MWCNTs nanocomposite.

By using elemental tests, EDX has verified that oxygen, copper, and carbon were used to create the nanocomposites. On a sample, mapping and scanning have been done in order to identify areas with higher concentrations of carbon and copper (**Figure 3**). The elemental map for the selected area, which is produced by the superposition of each element (C and Cu), is shown in the picture. Additionally, the nanocomposite’s oxygen, copper, and carbon contents have been identified. This is in favor of the creation of CuO/MWCNT nanocomposite.

### 3.2 Surface modification experiments

**Figure 4:**
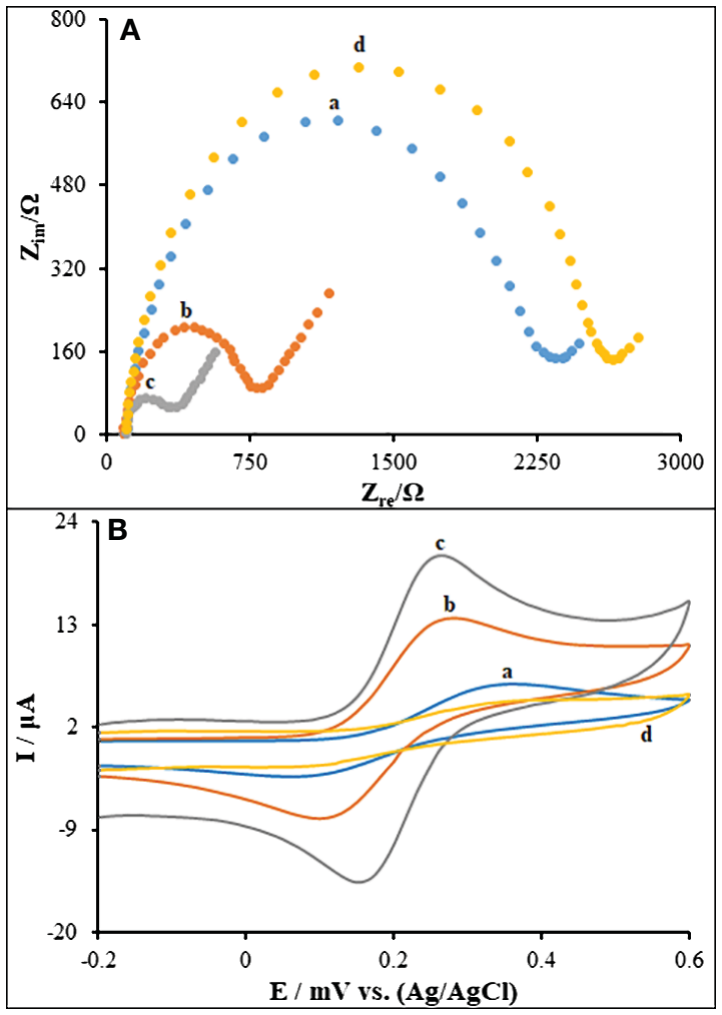
**(A)** Nyquist plots of **(a)** PA/PGE, **(b)** PA/MWCNTs/PGE **(c)** PA/CuO/MWCNTs/PGE, **(d)** ds-DNA/PA/CuO/MWCNTs/PGE in 0.1 M KCl containing 5.0 mM Fe(CN)_6_^3−/4−^. Frequency range 100 KHz to 0.1 Hz. **(B)** CVs of 5.0 mM [Fe(CN)_6_]^3-/4-^ in 0.1 M KCl: **(a)** PA/PGE, **(b)** PA/MWCNTs/PGE **(c)** PA/CuO/MWCNTs/PGE, **(d)** ds-DNA/PA/CuO/MWCNTs/PGE. Scan rate: 50 mV/s.

The modified PGE has been evaluated using electrochemical impedance spectroscopy (EIS) and cyclic voltammetry (CV). The Nyquist diagram in **Figure 4A** displays the PA/PGE (curve a), PA/MWCNTs/PGE (curve b), PA/CuO/MWCNTs/PGE (curve c) as well as the ds-DNA/PA/CuO/MWCNTs/PGE (curve d). The supporting electrolyte is 1.0 mM [Fe(CN)_6_]^−3/−4^ solution within 1.0 M of KCl. The existence of a conductive polymer layer upon PGE surface was blamed for a slight decrease in charge transfer resistance (R_ct_) (2348 ± 41 Ω). The R_ct_ values significantly decreased to 816 ± 12 Ω and 362 ± 23 Ω after the PA/PGE were modified with MWCNTs and CuO/MWCNTs, respectively demonstrating the high conductivity of MWCNTs and CuO for changing PA/PGE. Following ds-DNA immobilization upon the PA/CuO/MWCNTs/PGE surface (curve d), the R_ct_ value steadily increased (2649 ± 27 Ω). This result indicates that the electrode surface’s ability to transport electrons has been reduced as a result of ds-DNA’s non-conductive reaction that inhibited ferro/ferricyanide ions from getting to electrode (51).

As shown in **Figure 4B**, the CV patterns taken from the redox probe of Fe(CN)_6_^3-/4-^ respectively reveal some distinct redox peaks for PA/PGE (curve a), PA/MWCNTs/PGE (curve b), PA/CuO/MWCNTs/PGE (curve c) as well as the ds-DNA/PA/CuO/MWCNTs/PGE (curve d). The anodic and cathodic peak currents on the bare PGE (curve a) and PA/MWCNTs/PGE (curve b) were much lower than those on the PA/CuO/MWCNTs/PGE (curve c) with a greater peak-to-peak separation (ΔEp). This can be attributed to the nanocomposite components with better electrical conductivity owing to synergistic impact. The ds-DNA/PA/CuO/MWCNTs-modified PGE showed a reduction in the current response and an increase in the ΔEp value (curve d). The findings demonstrated the adsorption of negatively charged ds-DNA on cationic CuO/MWCNTs nanocomposite using electrostatic interaction, followed by repelling the Fe(CN)_6_^3-/4-^ ions with access to the electrode surface (52). Thus, the alterations of CV characteristics means ds-DNA immobilization on the modified electrode.

### 3.3 Guanine-Goserelin interactions at the surface of the sensor

**Figure 5:**
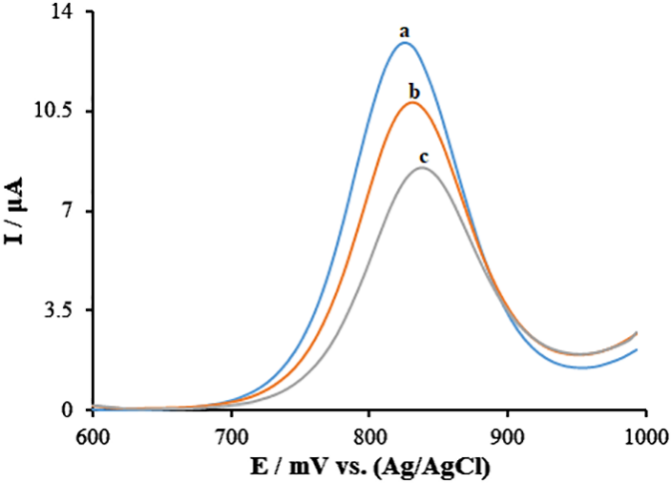
Differential pulse voltammograms of guanine after the interaction between 0.0, 10.0, and 30.0 μM Goserelin in ABS (0.5 M, pH 4.8) (curves a-c, respectively) and ds-DNA at ds-DNA/PA/CuO/MWCNTs/PGE.

In order to assess the contacts among the Goserelin and guanine bases of ds-DNA, differential pulse voltammetry (DPV) has been utilized. The findings (**Figure 5**) show that guanine bases may be oxidized at ds-DNA/PA/CuO/MWCNTs/PGE surface, resulting in a 12.74 μA oxidation current at ~831 mV (curve a). Under ideal circumstances, the guanine’s oxidation signal dropped to 10.81 μA (potential: 832 mV) and 8.51 μA (potential: 838 mV), respectively, following the contact with 10.0 and 30.0 μM of Goserelin (curve b and curve c). This represents the interaction between guanine and Goserelin as well as the inactivation of guanine bases. Additionally, Over time, the values of the oxidation potential shifted to the positive side, possibly as a result of Goserelin intercalations with minor grooves in ds-DNA.

### 3.4 Affinity of ds-DNA towards Goserelin

A stronger ds-DNA-Goserelin affinity implies higher sensitivity and selectivity of the biosensor. To obtain the affinity between ds-DNA-Goserelin, modified biosensor was incubated in different concentrations of the Goserelin target and related impedance signals were recorded in a 3.0 mM Fe(CN)63-/4- solution containing 0.1 M KCl. Based on the Equation 2:


(2) ds−DNA+n target Goserelin  ←Kd→Kads−DNA:n target Goserelin

at hybridization equilibrium, the association/dissociation constant can be expressed as (Equation 3):


(3) $${\text{K}}_{\text{a}}=\frac{\left[\text{ds}-\text{DNA}:\text{n target Goserelin}\right]}{\left[\text{ds}-\text{DNA}\right]\left[\text{n target Goserelin}\right]}\;$$

The parameters constant including, n; Hill coefficient, Ka; association constant, and Kd; dissociation constant (Kd=1Ka) can be obtained using a Hill plot based on the Equation 4 (53):


(4) $$\text{log}\frac{\text{Y}}{1-\text{Y}}=\text{log}\frac{1}{{\text{K}}_{\text{d}}}+\text{n}\log\left[\text{target Goserelin}\right]$$

Where, $\text{ Y}=\frac{\Delta {\text{R}}_{\text{ct}}}{\Delta {\text{R}}_{\text{ct},\text{max}}}$ and ΔRct and ΔRct, max represent the change and the maximum change in Rct, respectively.

**Figure 6:**
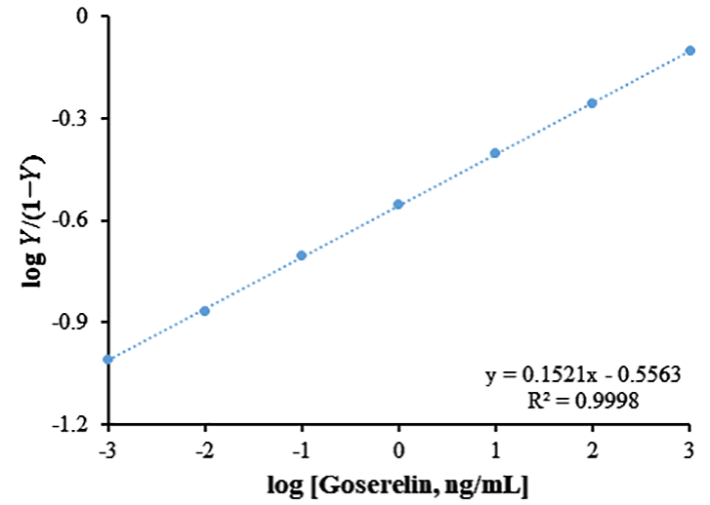
Hill plot; log *Y*/(1−*Y*) as a function of log [Goserelin] for ds-DNA, where Y is ΔR_ct_/ΔR_ct (max)_ and ΔR_ct_ is changing in charge transfer resistance.

Based on the Hill plot for Goserelin (**Figure 6**), the values of Kd, Ka, and n for this biosensor were 3.59 ng/mL, 0.28 ng/mL, and 0.15, respectively. The low Kd of this biosensor may be responsible for the higher affinity of the ds-DNA toward the Goserelin.

### 3.5 Optimization factors affecting guanine-Goserelin interactions

As these are major elements affecting the created DNA biosensor’s sensitivity, the impacts of the electrolyte type, ds-DNA concentration and temperature as well as contact duration on Goserelin/ds-DNA bonding have been adjusted.

**Figure 7:**
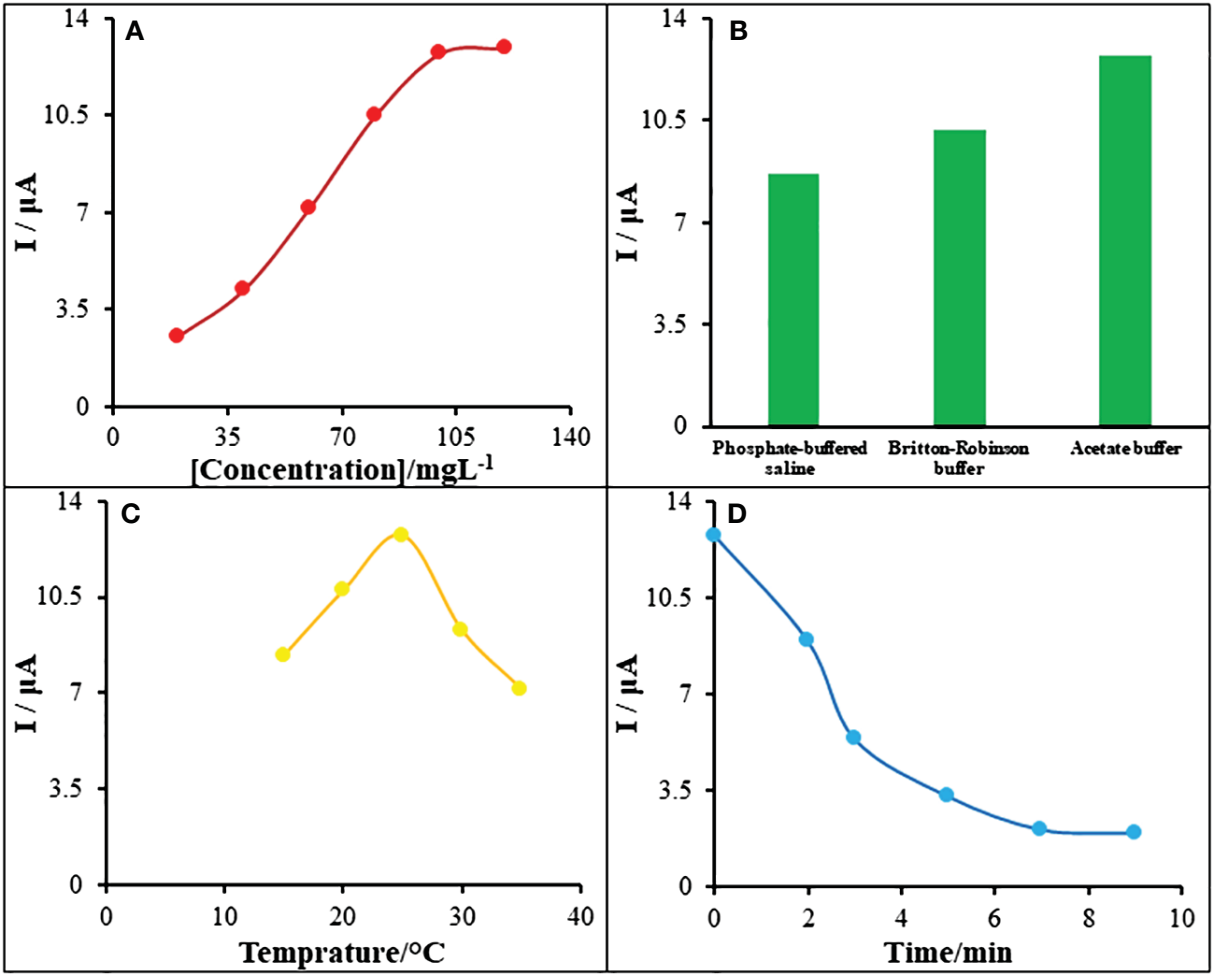
**(A)** The oxidation signal plot of guanine vs. ds-DNA concentration (20.0-120.0 mg/L). **(B)** Diagram of guanine oxidation current vs type of buffer solution. **(C)** Plot of guanine oxidation current vs intercalation solution temperature. **(D)** The influence of incubation time of 95.0 μM Goserelin in ABS (0.5 M, pH 4.8) on the response of ds-DNA/PA/CuO/MWCNTs/PGE.

The oxidation current of guanine increased when ds-DNA concentration grew from 20 mg/L to 120 mg/L, as shown by the plot of **Figure 7A**. The ds-DNA’s oxidation current has also been shown to remain steady when ds-DNA solutions were increased to 120 mg/L from 100 mg/L, proving that a solution of ds-DNA containing 100 mg/L covered the whole electrode surface. As a result, this value has been chosen as optimum conditions in following actions.

Additionally, the phosphate buffer solution (PBS), acetate buffer solutions (ABS) at pH = 4.8 and Britton-Robinson were used to record the guanine signal of 100 mg/L ds-DNA immobilized on the PA/CuO/MWCNTs/PGE surface in order to improve the electrolyte solution’s nature. The results as presented in **Figure 7B**, confirm that the maximum oxidation guanine signal could be observed using an acetate buffer, and this electrolyte was hence selected for further experiments.

In constructing the DNA biosensor along with enhancing its susceptibility, it is crucial to consider the contact temperature within ds-DNA/Goserelin bonding process. The optimal temperature was set at T = 25°C since the data in the **Figure 7C** indicated that the best contact occurred at this temperature. There was inadequate kinetic interaction among ds-DNA and Goserelin at lower temperatures and under the same conditions, and the bond is broken after forming at higher temperatures.

To reduce the amount of interaction time needed among ds-DNA and Goserelin, the ds-DNA oxidation signals at 100 mg/L when 95.0 μM Goserelin is present were also observed in various intercalation periods. According to the figure in **Figure 7D**, the interaction among the guanine in ds-DNA; Goserelin lowered the guanine’s oxidation current as the interaction period increased from 2.0 min to 9.0 min. When this period was extended to 7 or 9 minutes, the lower guanine signal persisted. This may be because of the active sites within ds-DNA becoming saturated. As a result, 7.0 minutes was chosen as the ideal setting for creating the biosensor.

### 3.6 Dynamic range and limit of detection

**Figure 8:**
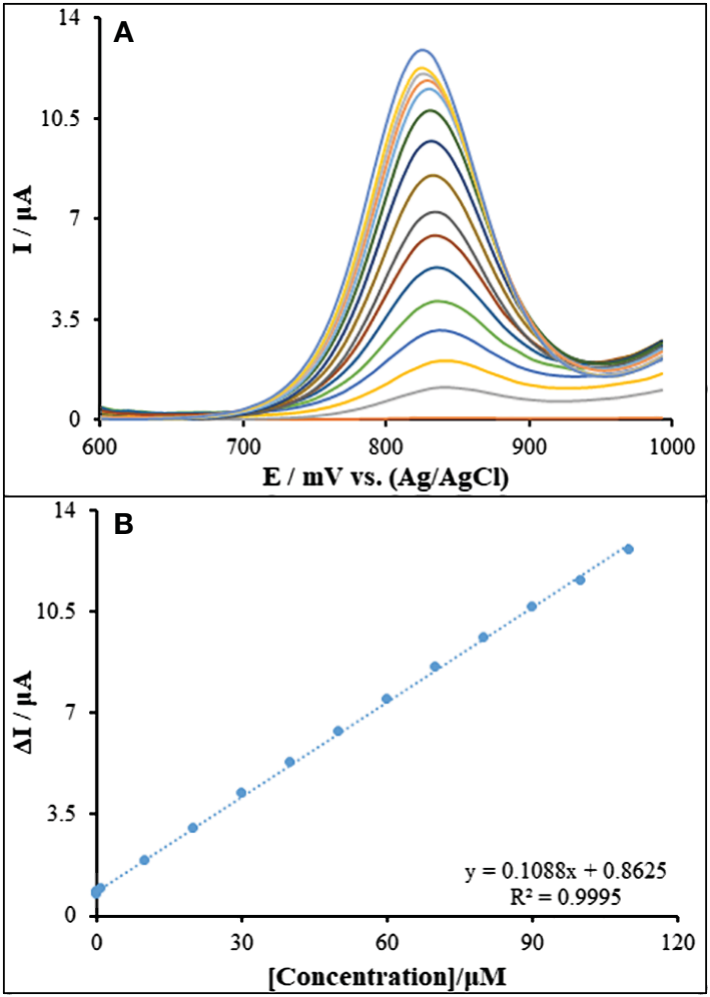
**(A)** Voltammograms of ds-DNA/PA/CuO/MWCNTs/PGE for different concentrations of Goserelin in ABS (0.5 M, pH 4.8). From top to bottom (1-16), 0.0, 0.001, 0.1, 1.0, 10.0, 20.0, 30.0, 40.0, 50.0, 60.0, 70.0, 80.0, 90.0, 100.0 and 110.0 μM. **(B)** Dependence of the net oxidation guanine current (different between guanine current in the absence and presence of Goserelin) vs. concentration of Goserelin.

The ΔI plot (the difference between the guanine current when Goserelin is present and when it is not) as an activity of Goserelin concentration is displayed in **Figure 8**. The **Figure 8** had a 0.21 nM limit of detection, a 0.1088 μA/μM sensitivity, and has been linear throughout the 0.001–110.0 μM Goserelin concentration range. This value of detection limit and the linear dynamic range for Goserelin observed for the ds-DNA/PA/CuO/MWCNTs/PGE are comparable and better than those obtained for several other previous studies (**Table 1**) (2, 7, 54, 55). When comparing with electrochemical methods, chromatography methods are expensive, sophisticated and multi-process techniques, with the need for sample preparation, pre-filtration and extraction as well as temperature monitoring.

**Table 1:** Comparison of major characteristics of various methods for the determination of Goserelin.

Methods	Dynamic ranges	Detection Limits	Ref.
Liquid chromatography–mass spectrometry	2.0-40.0 ng mL^-1^	2.0 ng mL^-1^	(2)
Reversed-phase high-performance liquid chromatography	2.0-90.0 µg mL^-1^	–	(7)
Liquid chromatography–electrospray ionization tandem mass spectrometry	0.01-30.0 ng mL^-1^	0.01 ng mL^-1^	(54)
Liquid chromatography–mass spectrometry	1.0-10.0 ng mL^-1^	1.0 ng mL^-1^	(55)
Voltammetry	0.001-110.0 µM	0.21 nM	This work

### 3.7 Reproducibility, stability of biosensor and interference study

**Figure 9:**
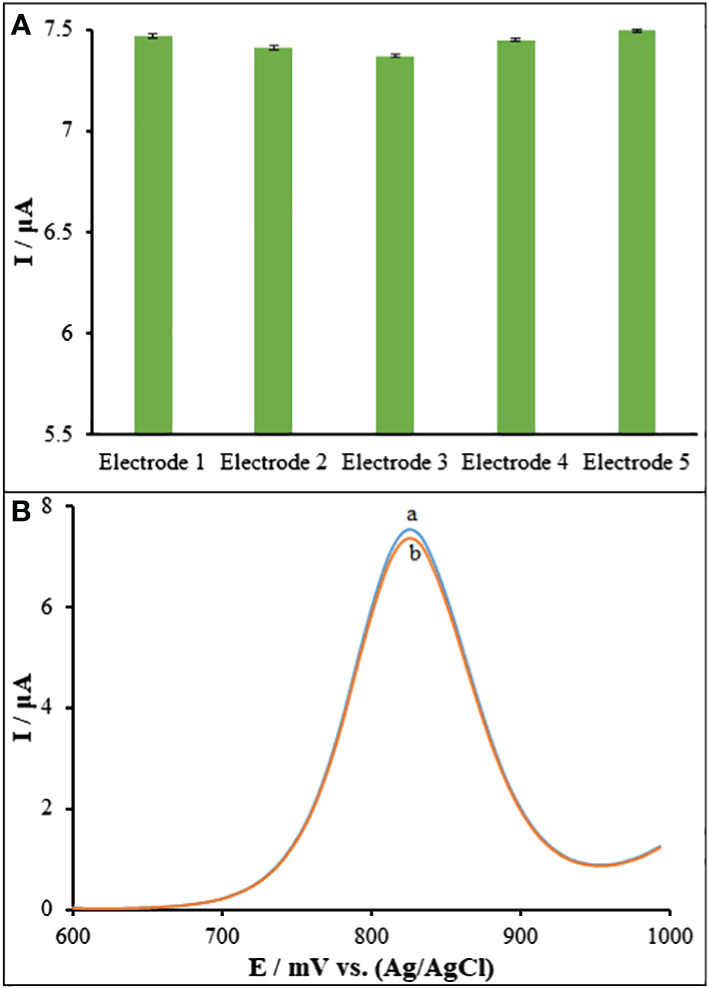
**(A)** DPVs of ds-DNA/PA/CuO/MWCNTs/PGE in ABS (pH=4.8) containing 40.0 μM Goserelin and (b) after 40 days. **(B)** Current responses of five ds-DNA/PA/CuO/MWCNTs/PGE fabricated under the same conditions.

**Figure 10:**
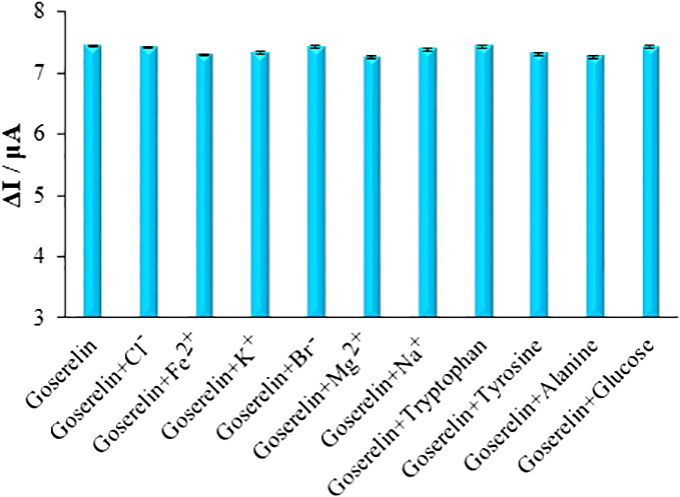
The columns are the current change of ds-DNA/PA/CuO/MWCNTs/PGE in ABS (pH=4.8) solution containing 40.0 μM Goserelin, Goserelin + 40.0 mM Cl^-^, Goserelin + 40.0 mM Fe^2+^, Goserelin + 40.0 mM K^+^, Goserelin + 40.0 mM Br^-^, Goserelin + 40.0 mM Mg^2+^, Goserelin + 40.0 mM Na^+^, Goserelin + 28.0 mM tryptophan, Goserelin + 28.0 mM tyrosine, Goserelin + 28.0 mM alanine and Goserelin + 28.0 mM glucose.

Five separate ds-DNA/PA/CuO/MWCNTs/PGEs have been utilized in recording the guanine signal in order to assess the reproducibility of sensor. A 7.44 μA solution of Goserelin (40.0 μM) showed an R.S.D. of 2.91% for the guanine signal, demonstrating the sensor’s high reproducibility (**Figure 9A**). The ds-DNA/PA/CuO/MWCNTs/PGE was also examined for the stability during 40 days. There was a reduction in the currents responses by 2.26% for Goserelin (40.0 μM) (**Figure 9B**). Accordingly, the as-developed DNA biosensor possessed a commendable reproducibility long stability for sensing Goserelin, so that it can show analytical application for detection of the Goserelin for over one month with no noticeable influence in its activity.

ds-DNA/PA/CuO/MWCNTs/PGE selectivity in analyzing the samples containing 40.0 μM Goserelin has been tested. Moreover, the findings showed that 1000-fold additions of Cl^-^, Fe^2+^, K^+^, Br^-^, Mg^2+^ and Na^+^ didn’t lead to any noticeable interference. Tryptophan, tyrosine, alanine, and glucose at 700-folds also did not exhibit any discernible influence (**Figure 10**).

### 3.8 Interday and intraday of biosensor

The intraday, 5 times, and interday, during 5 days, measurements was examined by emission recording the response of ds-DNA/PA/CuO/MWCNTs/PGE in the presence of 40.0 μM Goserelin. The observed *t* values of (2.17 and 1.24 for interday and intraday, respectively) were less than the critical *t* values = 2.776 in five replicate measurements (56), which confirmed there is no evidence of systematic error.

### 3.9 Analysis of real samples

For the purpose of testing the viability of ds-DNA/PA/CuO/MWCNTs/PGE for analyzing the injection and blood serum as well as urine samples were utilized as genuine samples. Further comparisons between the data and those in other studies (7) were made, as well as t-tests. The outcomes in **Table 2** demonstrate that ds-DNA/PA/CuO/MWCNTs/PGE can accurately analyze Goserelin in real samples. The results of the *t*-test in various concentrations (**Table 2**) supported that no significant systematic error existed in our analysis based on a comparison of the obtained *t* value and critical *t*-value = 2.776 in five replicate measurements. According to the recovery criteria (57, 58), and the values obtained from our method, as well as considering that each of the spike concentrations is in the confidence range, the recoveries are reasonable. These obtained results demonstrated the practicability of the proposed Goserelin sensing platform which is possible to apply even in complex matrices.

**Table 2:** Determination of Goserelin in real samples using ds-DNA/PA/CuO/MWCNTs/PGE (n = 5).

Sample	Detected (µM)	Added (µM)	Founded by proposed biosensor (µM)^a,b^	Founded by published method (7) (µM)^a,c^	Recovery (%)^b^	Recovery (%)^c^	*t*-test^b^	*t*-test^c^
Goserelin injection	8.7	2.0	10.5 ± 0.28	10.6 ± 0.29	98.1	99.1	1.59^e^	1.54^e^
		4.0	12.8 ± 0.29	12.4 ± 0.31	100.8	97.6	0.77^e^	2.16^e^
Human blood serum	ND^d^	6.0	5.9 ± 0.21	6.2 ± 0.24	98.3	103.3	1.06^e^	1.86^e^
		8.0	7.8 ± 0.32	7.9 ± 0.28	97.5	98.70	1.39^e^	0.79^e^
Urine	ND^d^	10.0	10.2 ± 0.20	10.1 ± 0.19	102.0	101.0	2.23^e^	1.17^e^
		12.0	11.9 ± 0.27	11.7 ± 0.25	99.2	97.5	0.82^e^	1.78^e^

a Mean ± standard deviation for n = 5.

b Proposed method.

c Published method (7).

d Not detect.

e P<0.05, 95%.

### 3.10 Molecular docking investigation

**Figure 11:**
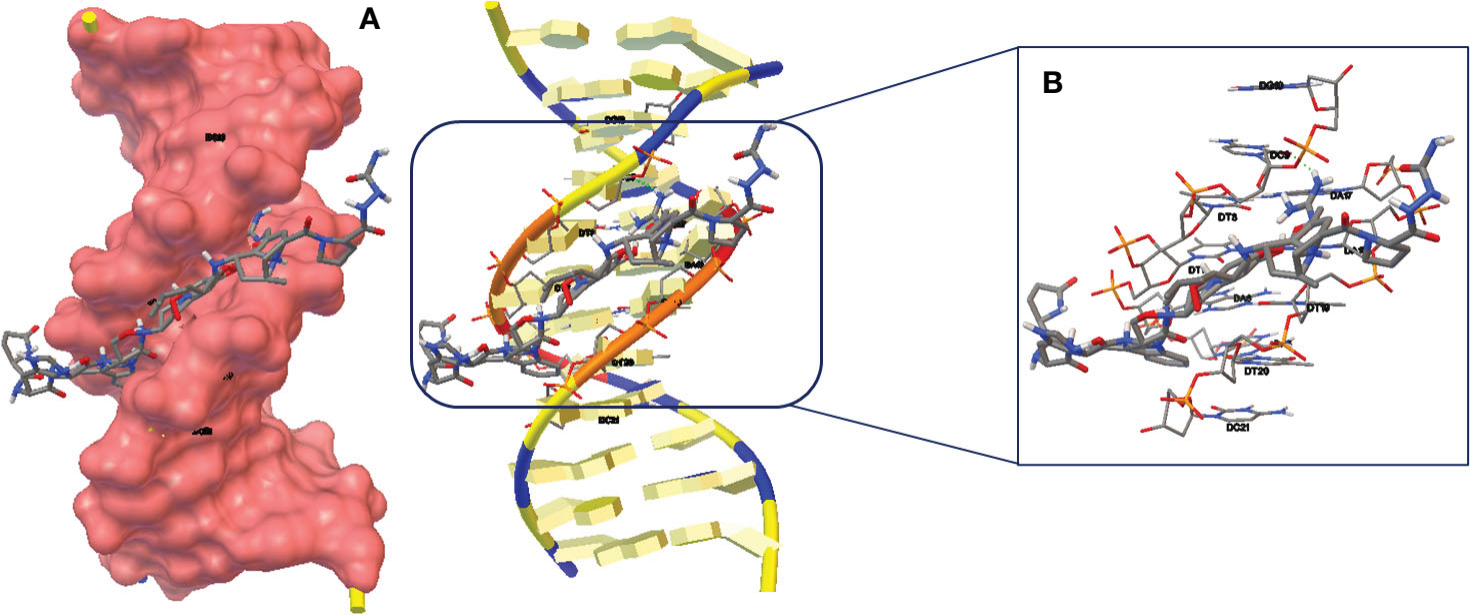
**(A)** Goserelin-DNA intercalation interaction. **(B)** Geometrical disposition of Goserelin in DNA intercalation.

The molecular docking investigation (**Figure 11**) used DNA fragment sequences, namely hexamer d(CGATCG)2 with an intercalation gap (PDB ID: 1Z3F) to estimate the Goserelin’s binding mechanism. **Figure 11** show the structure of DNA-Goserelin complexes. The results of the docking study showed that Goserelin intercalated into nitrogenous cytosine and guanine base pairs of the DNA receptor. The Goserelin-DNA complex is stabilized by π-π stacking interactions as well as intermolecular hydrogen bonds (HBs) with a binding energy of -14.82 kcal mol^-1^ and K_i_ 13.71 pM. It was discovered that the hydrogen atom in the Goserelin drug functioned as donor moieties in the formation of one O⋯H-N conventional hydrogen bond (HB) with the DNA base pairs. Hydrogen bond included: H (H_2_N) of Goserelin interacted with oxygen 1 (OP1) of thymine 4 (DT4).

### 3.11 Potential impact of biosensors and perspectives

The clinical practice of detecting pharmaceutical drug concentrations in patients’ biofluids at predetermined intervals to enable prompt and accurate dose management is known as therapeutic drug monitoring (TDM) (59). This procedure enables prompt medical intervention in the event of toxicity-related problems and/or dose modification to better meet therapeutic needs. Since antineoplastic medications typically exhibit a narrow range of efficacy between non-efficacy and toxicity, which is typically characterized by significant side effects, it becomes especially crucial in the treatment of cancer disorders (60).

An initial dose schedule is decided upon and TDM starts after the clinical circumstances of the patients are evaluated together with individual factors, such as weight, age, and other concurrent pharmacological regimens. Then, it becomes evident that each patient’s therapy is unique, necessitating customized medication delivery. As a result, clinical medicine has a difficult problem in providing real-time tailored therapy. In actuality, the secret to achieving this objective is quick and accurate diagnosis. Technological developments in the fields of biosensors and nanosciences provide a rare chance to address the problems listed above and get around the disadvantages brought on by expensive and time-consuming procedures (61).

In light of point-of-care testing, biosensors are useful due to their miniaturization, portability, quick analyses, simplicity of use, and inexpensive production costs. Few works address TDM, despite the fact that a sizable number discuss the application of biosensors in clinical chemistry and drug development (62).

The burgeoning interest in so-called nanobiosensors is largely due to their potential to deliver cutting-edge technology and instruments with unparalleled capabilities. While there are still issues that need to be resolved before these devices are developed and used in clinical settings, a number of analytical platforms have surfaced recently with encouraging outcomes (63). In order to improve patient outcomes and reduce laboratory costs, the scientific community is working to deploy such detection methods and create new technologies that will assist close the gap between accurate and timely analyses and successful individualized medication (64).

Nonetheless, there is little doubt that in the very near future, new instruments for clinical and point-of-care testing, as well as for drug development, will be made possible by nanobiosensors due to the progress made in nanoscience and micro/nanofabrication technologies.

## 4 Conclusion

The study concentrated on the creation of DNA biosensors with high sensitivity for the evaluation of Goserelin as a cancer treatment. In order to do this, a DNA biosensor was used and a pencil graphite electrode has been modified with ds-DNA, polyaniline, as well as CuO/MWCNTs nanocomposite. With Goserelin present or absent at concentrations between 0.001 and 110.0 μM, the guanine signals were used to examine this anticancer drug. The binding of Goserelin and DNA structure was verified by docking theoretical experiments. Finally, the combination of ds-DNA, PA, CuO, MWCNTs, and PGE shown excellent performance for the detection of Goserelin in real samples.

## Data availability statement

The original contributions presented in the study are included in the article/supplementary material. Further inquiries can be directed to the corresponding author.

## Ethics statement

The studies involving humans were approved by Ethical Committee of King Khalid University. The studies were conducted in accordance with the local legislation and institutional requirements. The participants provided their written informed consent to participate in this study.

## Author contributions

LL: Conceptualization, Formal analysis, Writing – review & editing. FA: Validation, Writing – original draft. AA: Writing - reviewing & editing, Methodology, Data curation. GS: Methodology, Writing – review & editing. MM: Validation, Writing – review & editing. AA: Data curation, Writing – original draft. AA: Supervision, Writing – original draft.
